# Global Identification of EVI1 Target Genes in Acute Myeloid Leukemia

**DOI:** 10.1371/journal.pone.0067134

**Published:** 2013-06-27

**Authors:** Carolyn Glass, Charles Wuertzer, Xiaohui Cui, Yingtao Bi, Ramana Davuluri, Ying-Yi Xiao, Michael Wilson, Kristina Owens, Yi Zhang, Archibald Perkins

**Affiliations:** 1 Department of Pathology and Lab Medicine, University of Rochester Medical Center, Rochester, New York, United States of America; 2 Molecular and Cellular Oncogenesis Program, Center for Systems and Computational Biology The Wistar Institute, Philadelphia, Pennsylvania, United States of America; 3 Department of Pathology, Yale University School of Medicine, New Haven, Connecticut, United States of America; West Virginia University School of Medicine, United States of America

## Abstract

The ecotropic virus integration site 1 (EVI1) transcription factor is associated with human myeloid malignancy of poor prognosis and is overexpressed in 8–10% of adult AML and strikingly up to 27% of pediatric MLL-rearranged leukemias. For the first time, we report comprehensive genomewide EVI1 binding and whole transcriptome gene deregulation in leukemic cells using a combination of ChIP-Seq and RNA-Seq expression profiling. We found disruption of terminal myeloid differentiation and cell cycle regulation to be prominent in EVI-induced leukemogenesis. Specifically, we identified EVI1 directly binds to and downregulates the master myeloid differentiation gene *Cebpe* and several of its downstream gene targets critical for terminal myeloid differentiation. We also found EVI1 binds to and downregulates *Serpinb2* as well as numerous genes involved in the Jak-Stat signaling pathway. Finally, we identified decreased expression of several ATP-dependent P2X purinoreceptors genes involved in apoptosis mechanisms. These findings provide a foundation for future study of potential therapeutic gene targets for EVI1-induced leukemia.

## Introduction

### Evidence for the Role of EVI1 in Myeloid Leukemia

The ecotropic virus integration site 1 (EVI1) is an oncogenic transcription factor associated with human myeloid malignancy and several solid epithelial cancers [1], [2], [3]. Aberrant EVI1 expression occurs in 8–10% of human adult acute myeloid leukemia (AML) and strikingly up to 27% of pediatric mixed lineage leukemia (MLL) rearranged leukemias [4]. EVI1 is one of several protein isoforms encoded by the *MECOM* locus at human chromosome 3q26 which also yields the MDS1 and MDS-EVI1 protein isoforms [5]. The role of MDS1 and MDS-EVI1 in malignancy is still unclear, while the EVI1 transcription factor, specifically the 135kDa isoform has been reported as a malignant contender [6]. EVI1 overexpression in human AML most frequently occurs with rearrangements at chromosome 3q26 [7], [8]. The MLL-AF9 fusion oncoprotein has also been shown to activate the *MECOM* locus in the setting of AML [9].

Although previous studies have certainly supported the role of EVI1 in myeloid malignancy, establishing an experimental system with consistent disease induction has been challenging. Forced expression of *Evi1* in murine lineage-negative bone marrow (BM) cells via retroviral transduction followed by transplantation back into irradiated recipients has yielded conflicting results. Buonamici et al demonstrated *Evi1* transduced BM in C57BL6 recipients developed lethal myelodysplastic syndrome (MDS) 8–12 months after bone marrow transplantation (BMT), but none developed AML [10]. In another study, Cuenco et al showed none of the mice that received BM cells transduced with the *Evi1* retrovirus developed AML [11]. In contrast to these results, Yoshimi et al showed C57BL6 mice transplanted with *Evi1*-transduced bone marrow cells all developed AML (characterized by high percentage of blasts on BM smear, positive myeloid markers for leukemic cells, marked splenomegaly) and died within 6–11 months after BMT [12]. Furthermore, a separate study demonstrated *Evi1* does not induce AML alone, but requires co-expression with *Hoxa9/Meis1* to drive leukemogenesis [13]. Collectively, the current data does not support a specific experimental approach by which *Evi1* overexpression by itself consistently induces leukemogenesis.

### *EVI1* Binds DNA to Induce Leukemic Transformation

The *Evi1* gene spans 65 kb of genomic DNA with 16 exons which generate 3 different isoforms (135kDa [14], 123kDa [15], 103kDa [16], [17]). The 135kDa and 123kDa isoforms both contain two zinc finger domains, ZF1 and ZF2 that bind DNA in a sequence specific manner [18], [19]. The 103kDa isoform lacks ZF1 domain fingers 6 and 7, and fails to bind DNA via that domain [16]. We previously demonstrated ZF1 binds to the motif GACAAGATA with high affinity and specificity in vitro [19] and showed ZF1, but not ZF2 is critical for malignant activity [20], [21]. Zhang et al recently demonstrated ZF1 DNA binding can be inhibited with a pyrrole-imidazole polyamide with high specificity and affinity [20].

Several studies have identified EVI1 downstream target genes associated with putative leukemogenic functions [22], [23], [24], [25], [26], [27], [28], [29]. Direct EVI1 binding to the promoter of *Gata2*, an essential regulator of HSC proliferation [30], was demonstrated by ChIP-qPCR. *Gata2* has been reported to be aberrantly expressed in 87% of de novo AML cases [31]; our analysis of RNA expression data from AML patients shows a good correlation between EVI1 and GATA2 expression (Pearson correlation (r) of 0.42–0.52; unpublished data). However a definitive requirement for *Gata2* in EVI1-induced leukemogenesis has yet to be shown. A genome wide transcription factor binding study for EVI1 has been reported recently for a human ovarian cancer cell line [28]. The study demonstrated over 25% of EVI1-occupied genes were also bound by activator protein 1 (AP1), providing evidence for a synergistic cooperative interaction between EVI1 and AP1, specifically the FOS protein. AP1 controls important cellular processes such as apoptosis, cellular differentiation and proliferation and has been described as a “nuclear decision-maker” critical for determining life or death cell fate decisions [32]. Taken together, these studies provide evidence that EVI1 directly binds critical genes associated with malignant transformation.

### Biologic Effects of *EVI1*

AML cells harbor dysfunction of one or more of the following decision processes: cellular differentiation, programmed cell death (apoptosis) and cellular growth control. In regards to differentiation, EVI1-induced leukemic cells have been associated with defects in terminal myeloid differentiation, specifically disruption of granulocytic and erythroid commitment [33], [34]. Morishita et al first reported *Evi1* overexpression in 32Dc13 myeloid cells inhibits terminal differentiation to granulocytes in response to granulocyte-colony stimulating factor (G-CSF) [34]. However it was later shown that native 32Dc13 cells harbor a proviral insertion at *Evi1* and overexpress both mRNA and protein [35]. In addition, this assay is difficult to interpret, since the EVI1-overexpressing cells undergo cell death upon treatment with G-CSF. Another study showed *Evi1* overexpression in BM progenitors result in impaired myeloid terminal differentiation associated with a subset of genes regulated by PU.1 binding [36]. More recently, *Evi1* has been shown to be preferentially expressed in HSCs and required for the maintenance of hematopoiesis [37], [38]. However, there is still a paucity of data connecting EVI1 binding to specific gene targets and how it influences definitive hematopoietic cell lineage decisions.

In addition to blocked differentiation, *Evi1* leukemic cells also demonstrate resistance to apoptosis which has been associated with ineffectiveness of chemotherapy regimens, high relapse rates and poor prognosis [39]. The survival advantage conferred by *Evi1* in myeloid leukemic cells has been well studied [40], [41], [42]. Kurokawa et al showed EVI1 directly interacts with and inhibits c-Jun N-terminal kinase (JNK) to protect cells from JNK-activated stress-induced cell death [42]. EVI1 ZF1 also binds and activates the *BCL-XL* promoter in the colon carcinoma HT-29 cell line overexpressing EVI1, resulting in inhibition of apoptosis [40]. However, a role for the deregulation of JNK and BCL-XL in leukemogenesis has not been directly addressed. We have also shown that *Evi1* knockdown in DA-1 leukemic cells induces apoptotic features such as DNA fragmentation, reduction in mitochondrial membrane potential and cleavage of procaspases 3 and 9 (delCampo et al, in revision). Previous studies demonstrate a single amino acid mutation (R205N) in ZF1 prevents EVI1 binding to DNA [23]. Preliminary data shows DA-1 leukemic cells overexpressing the R205N mutant EVI1 exhibit significantly increased apoptosis, supporting the notion that ZF1 DNA binding is critical in suppressing apoptosis (del Campo et al, in revision). Collectively, there appears to be good evidence for EVI1-induced anti-apoptosis mechanisms, but additional studies are needed to confirm these findings and to flesh out the precise mechanism.

Finally, inappropriate *Evi1* expression has been associated with aberrant cell cycle regulation resulting in excessive proliferation [43], [44], [45]. Abnormal cellular proliferation mediated by the TGFβ pathway has frequently been cited in *Evi1* expressing cells. EVI1 has been reported to interact with and repress SMAD3 function, resulting in loss of TGFβ- induced antiproliferative effects [43]. However, the relevance of this to AML is not clear. *Evi1* has also been shown to accelerate the cell cycle of Rat-1 fibroblasts [45], murine 32Dcl3 myeloid cells [44] and murine embryonic stem cells [46]. However according to other reports, the cell cycle and proliferative activity of HEL cells is not influenced by EVI1 overexpression [47]. These conflicting data seem to indicate that EVI1-regulated proliferative effects in AML have yet to be elucidated.

Various other biologic functions regulated by EVI1 downstream gene targets have also been identified by ChIP assay and confirmed by PCR experiments. These functions include disruption of normal hematopoiesis [48], growth arrest in response to stressful stimuli [27], calreticulin function [24], and microRNA gene silencing [26].

Despite these many findings, a cohesive mechanism by which *Evi1* induces leukemogenesis remains elusive.

Here, we report for the first time ChIP-Seq combined with RNA-Seq expression profiling in *Evi1*-overexpressed leukemic cells. We found that deregulation of genes involving apoptosis, differentiation and proliferative mechanisms likely all contribute to the development of *Evi1* leukemogenesis. Specifically, we identified EVI1 directly binds to and downregulates a master myeloid differentiation regulator gene, *Cebpe*, in both *Evi1* overexpressed leukemic cell lines. We found a high number of downstream gene targets of *Cebpe* were also downregulated in EVI1 leukemic cells. We also identified EVI1 binds to and deregulates *Serpinb2* as well as numerous genes involved in the Jak-Stat signaling pathway to drive cellular differentiation. Finally, we found several ATP-dependent P2X purinoreceptors involved in apoptosis mechanisms, particularly *P2rx7*, to be significantly downregulated.

## Results

### Whole Transcriptome Analysis using RNA-Seq

To identify genes differentially expressed between *Evi1* overexpressed (DA-1, NFS-60) and in shRNA *Evi1* knockdown cells, RNA was extracted to generate transcriptome-wide expression profiles. Genes with expression levels significantly greater or reduced relative to the control shRNAs (shLuc and shScr) cell lines have been termed upregulated and downregulated, respectively. High throughput parallel sequencing using the Illumina Genome Analyzer IIx revealed 806 significantly deregulated (p<0.05) genes in DA-1 cells (481 upregulated, 325 downregulated in the *Evi1* overexpressed cells compared to the *Evi1* shRNA knockdown) and 782 deregulated genes in the NFS-60 cell line (437 upregulated, 345 downregulated, Dataset S1).

To gain further insight into biological pathways associated with the significantly up or downregulated genes identified in EVI1-induced leukemia, analysis using the Database for Annotation, Visualization and Integrated Discovery (DAVID) [49] bioinformatics tool was performed (Table 1). In DA-1 EVI1 leukemic cells, significantly upregulated genes were enriched for KEGG pathways involving hematopoietic cell lineage (myeloid, but not lymphoid p = 7.5E^−3^) and cytokine-cytokine interaction (p = 6.7E^−2^). Significantly downregulated DA-1 genes were enriched for pathways involving cytokine-cytokine receptor interaction (p = 9.4E^−3^), Mapk signaling (p = 8.3E^−2^), Jak-Stat signaling, and hematopoietic cell lineage (p = 8.7E^−2^). In NFS-60 EVI1 leukemic cells, significantly upregulated genes (i.e., higher in EVI1-expressing cells) were enriched for KEGG pathways involving hematopoietic cell lineage (p = 7.1E^−3^) and pathways in cancer (p = 4.3E^−2^). Significantly downregulated NFS-60 genes were enriched for cytokine-cytokine receptor interaction (p = 6.6E^−6^), Jak-Stat signaling (p = 1.2E^−2^), and chemokine signaling (p = 1.4E^−2^).

**Table 1:** Significantly enriched KEGG pathways for genes with aberrant expression levels in EVI1 leukemic cells.

Enriched KEGG Pathway	DA-1 Upregulated Genes	NFS-60 Upregulated Genes	DA-1 Downregulated Genes	NFS-60 Downregulated Genes
Chemokine signaling				x
Cytokine-cytokine interaction	×		×	×
Hematopoietic cell lineage	×	×	×	
Jak-Stat signaling			×	×
Mapk signaling			×	
Pathways in cancer		×		

DAVID analysis was performed for significantly upregulated and downregulated genes in both the *Evi1* overexpressing DA-1 and NFS-60 leukemic cell lines.

**Figure 1:**
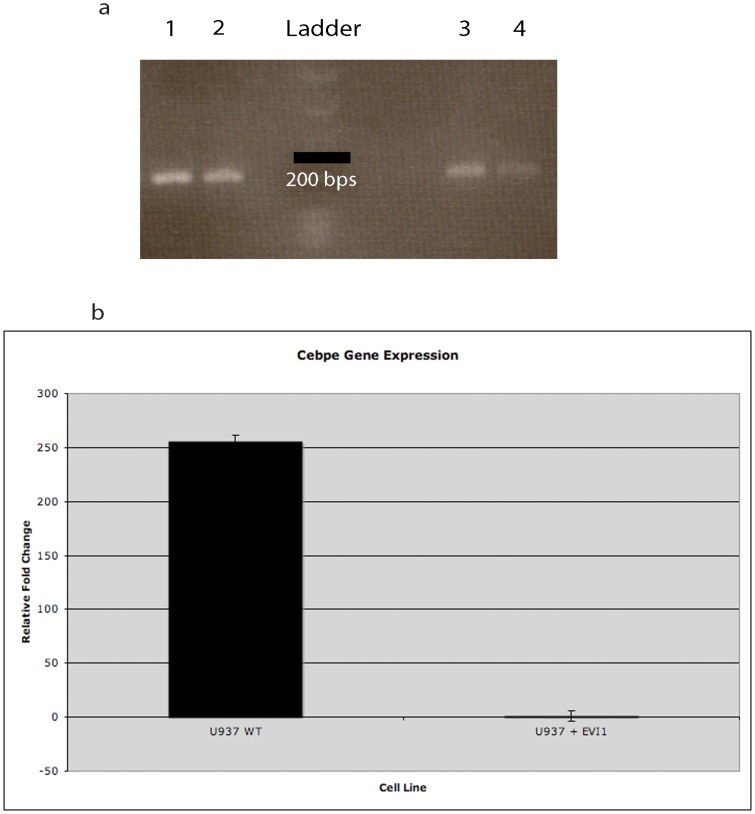
Significant downregulation of *Cebpe* in human *Evi1* overexpressed leukemic cells. **a)** Lanes 1 and 2 are beta-actin positive controls for U937 wildtype and U937+*Evi1* cells, respectively. Lane 3 sample is U937 wildtype cells (without *Evi1* overexpression) and Lane 4 sample is U937 with *Evi1* overexpression. *Cebpe* is downregulated in EVI1 overexpressed U937 human leukemic cells (Lane 4). **b)** Quantitative RT-PCR shows significant downregulation of *Cebpe* in *Evi1* overexpressed human leukemic cells. The y-axis value denotes the relative levels of RNA expression based on normalized Ct values. U937+*Evi1* cells had 8 point increase in Ct value (or 256 fold decrease) compared to the U937 wildtype cells (p<0.001).

**Figure 2:**
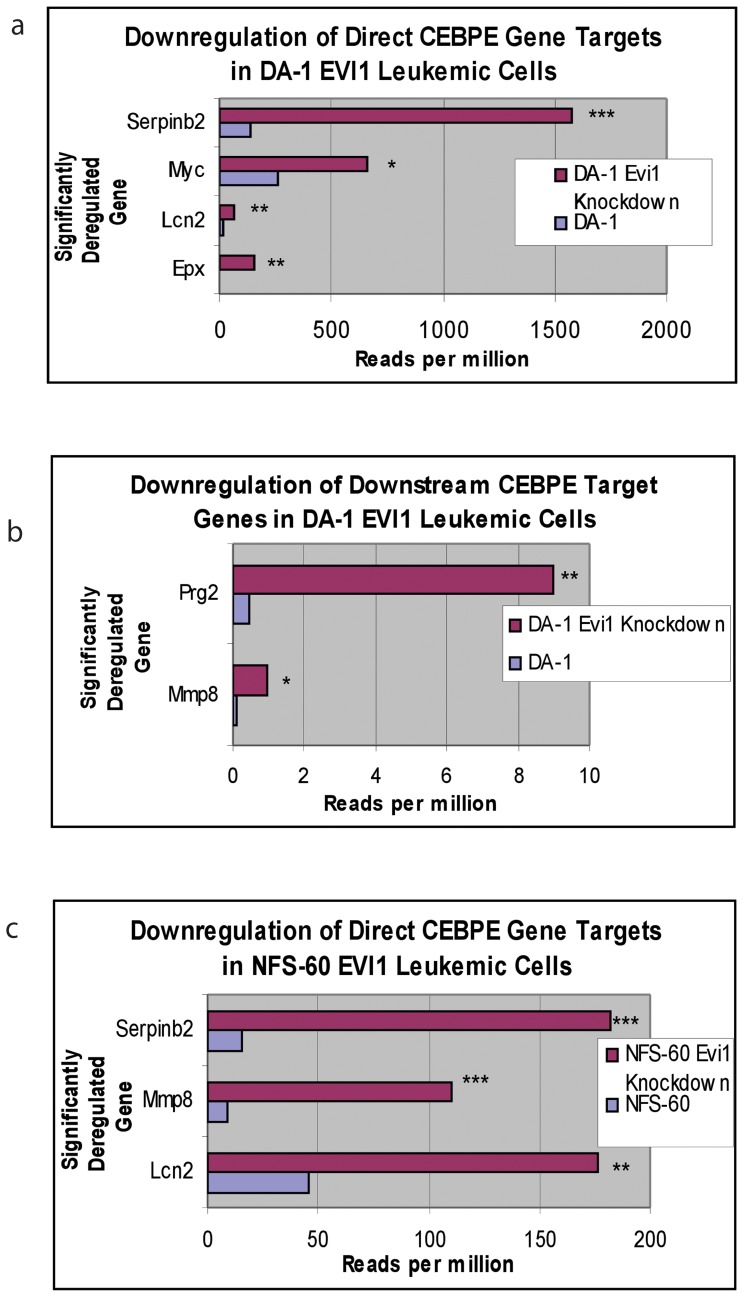
Direct downstream gene targets of the C/EBP-ε transcription factor. Several C/EBP-ε target genes were significantly downregulated in both *Evi1* overexpressed leukemic cell lines. The x-axis is the significantly downregulated gene and the y-axis denotes reads per million on RNA-Seq analysis. **a)** In DA-1 *Evi1* overexpressed cells, *Serpinb2* had an 11.4-fold decrease (p = 0.0006), *Myc* had a 2.5-fold decrease (p = 0.04), *Lcn2* had a 3.4-fold decrease (p = 0.001), *Epx* had a 13.5-fold decrease (p = 0.0003). **b**) In DA-1 *Evi1* overexpressed cells, although the overall level of transcription for *Mmp8* and *Prg2* were reduced compared to the other significantly downregulated C/EBP-ε gene targets, they exhibited a very large significant fold change in the EVI leukemic cells. *Mmp8* had a 13.5-fold decrease (p = 0.02), and *Prg2* had a19.0-fold decrease (p = 0.003) in the EVI1 leukemic cells compared to the knockdown. **c)** In NFS-60 *Evi1* overexpressed cells, *Lcn2* had a 3.9-fold decrease (p = 0.001), *Mmp8* had a 12.7-fold decrease (p = 6.5E^−5^), and *Serpinb2* had an 11.5-fold decrease (p = 5.8E^−5^).

A total of 35 genes were significantly upregulated and 42 genes were significantly downregulated in both cell lines (Table 2). We identified a 2-fold downregulation of *Cebpe*, a master regulator of terminal myeloid differentiation, in both the murine EVI1 leukemic cell lines. However significance was only reached in the NFS-60 cell line due to the low number of RNA-Seq reads in the DA-1 cell line for the *Cebpe* gene (Dataset 1). A U937 human leukemic cell line with *Evi1* overexpression via retroviral infection also confirmed significant downregulation of *Cebpe* by PCR (Figure 1a). We also found a high number (N = 6) of significantly downregulated direct gene targets of C/EBP-ε in DA-1 leukemic cells (Dataset S2, Figure 2a and 2b). In NFS-60 leukemic cells, 3 C/EBP-ε direct gene targets were also significantly downregulated (Dataset S3, Figure 2c). These results demonstrate EVI1 leukemic cells not only exhibit downregulation of *Cebpe* expression, but also suppression of downstream target genes of the master differentiation regulator.

**Table 2:** Significantly deregulated genes based on RNA-Seq demonstrated in both murine cell lines (DA-1 and NFS-60).

Significantly Upregulated Gene in Both Cell Lines (N = 35)	Significantly Downregulated Gene in Both Cell Lines (N = 42)
Asph	Acsbg1
Cenpe	Anxa3
Ces2g	Aqp9
Cmpk2	Bcat1
Ddx60	Car12
Eif2ak2	Ccl4
Enpp5	Ccl6
Fam46b	Ccl9
Gp1ba	Cpa3
Gp9	Ctla2a
Igf1r	Ctsg
Ltb	Cyp11a1
Lyst	Dgat2
Mecom (Evi1)	Fcgr2b
Mfsd2b	Fcgr3
Mki67	Fgf21
Msrb3	Fos
Nmnat2	Gas7
Nox1	Gng10
Oas2	Gpr34
Plekha6	Hif1a
Plekhg5	Hrk
Scarf1	Ifitm1
Serpini1	Ifitm6
Slc6a20a	Il1rn
Smpdl3b	Lcn2
Sox6	Lif
Spns2	Mcpt8
Tlr1	Mmp8
Tmod1	Olr1
Treml2	Osm
Ube1l	P2rx3
Utrn	Reep6
Zcchc24	Saa3
Zfpm1	Serpinb2
	Serpinf1
	Slamf9
	Slpi
	Socs1
	Srm
	Svip
	Tph1

**Figure 3:**
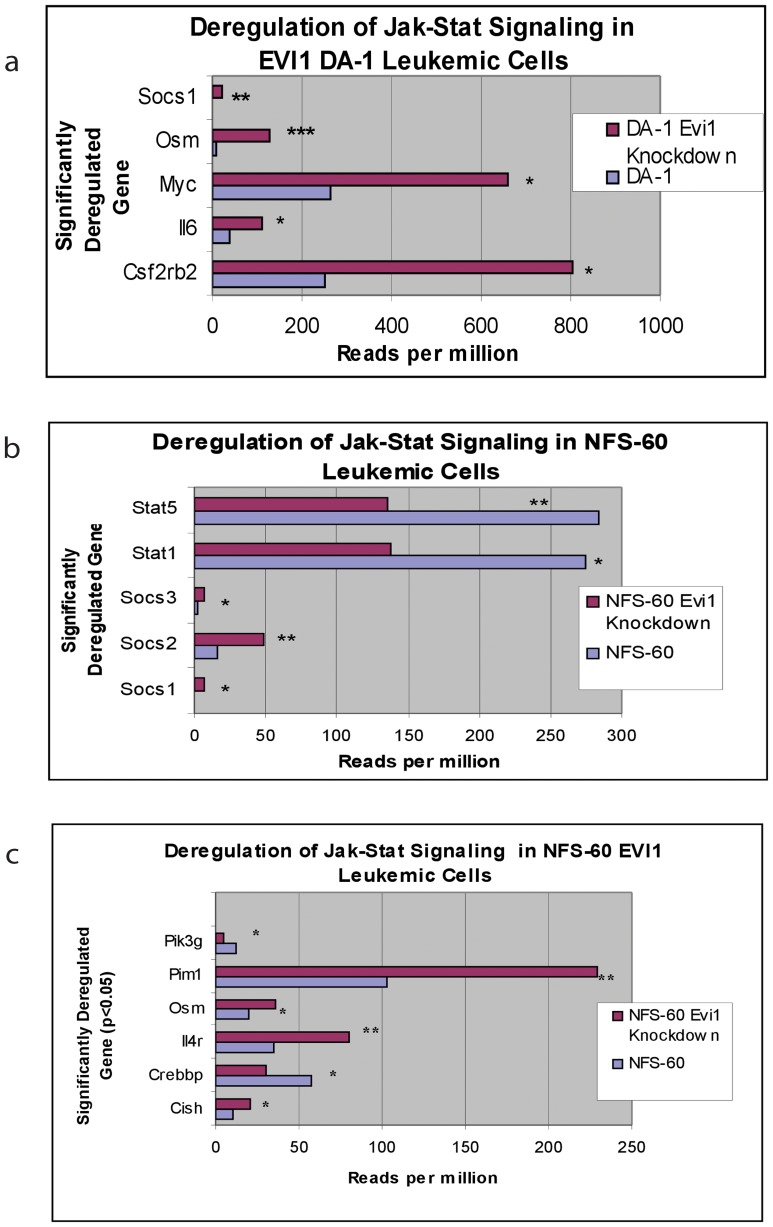
Significant deregulation of the Jak-Stat signaling pathway. Numerous genes involved in the Jak-Stat pathway were found to be aberrantly expressed in both the EVI1 leukemic cell lines. The x-axis is the significantly downregulated gene and the y-axis denotes reads per million on RNA-Seq analysis. **a**) In DA-1 *Evi1* overexpressed cells, *Socs1* had a 5.7-fold decrease (p = 0.001), *Osm* had a 13-fold decrease (p = 0.0003), *Myc* had a 2.5-fold decrease (p = 0.04), *Il6* had a 2.8-fold decrease (p = 0.02), *Csf2rb* had a 3.2-fold decrease (p = 0.02). **b)** In NFS-60 *Evi1* overexpressed cells, *Stat5* had a 2.1-fold increase (p = 0.01), *Stat1* had a 2.0-fold increase (p = 0.02), *Socs3* had a 3.7-fold decrease (p = 0.03), *Socs2* had a 3.0-fold decrease (p = 0.01), *Socs1* had a 4.5-fold decrease (p = 0.02). **c)** In NFS-60 *Evi1* overexpressed cells, *Pik3c2g* had a 2.8-fold increase (p = 0.04) *Pim1* had 2.2-fold decrease (p = 0.01), *Osm* had a 1.8-fold decrease (p = 0.04), *Il4r* had a 2.3-fold decrease (p = 0.01), *Crebbp* had a 1.9-fold increase (p = 0.05), *Cish* had a 2.0-fold decrease (p = 0.05).

**Figure 4:**
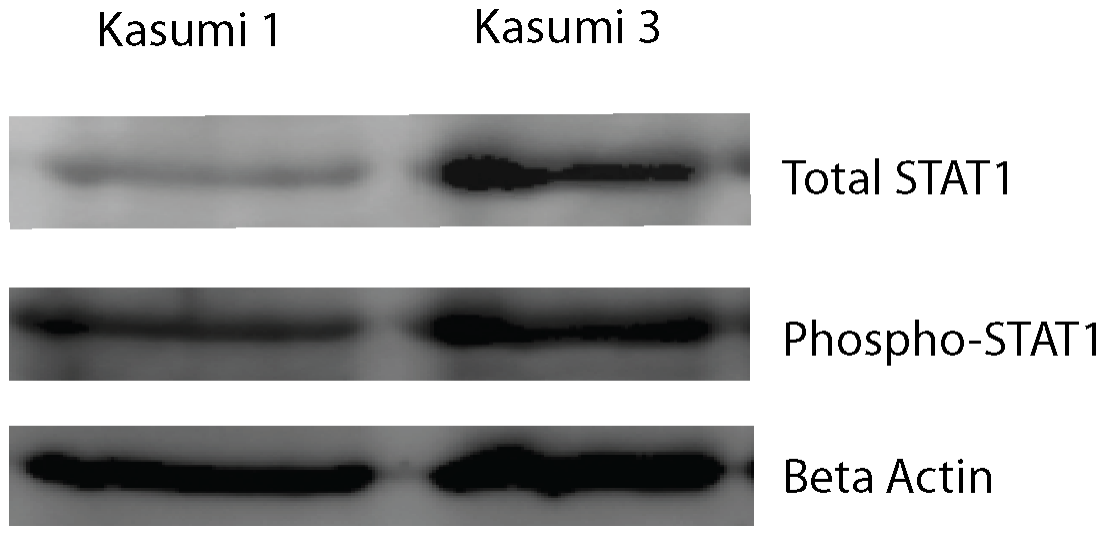
Increased endogenous STAT1 phosphorylation in human *Evi1* overexpressed leukemic cell lines. **a)** Western blot analysis using anti-total-STAT1 antibody. Lane 1 from left shows total STAT1 protein expression level in Kasumi 1 cells. Lane 2 shows total STAT1 protein level in Kasumi 3 cells. *Evi1* overexpressed myeloid leukemic cells demonstrate a higher baseline of STAT1 protein, consistent with our mRNA findings. **b)** Western blot analysis using anti-phospho-STAT1 antibody. Lane 1 from left shows endogenous phosphorylated STAT1 protein expression level in Kasumi 1 cells (human leukemic cell line without *Evi1* expression. Lane 2 shows the STAT1 protein level in Kasumi 3 cells. **c)** Beta actin loading control.

Expression levels of numerous genes associated with the regulation of Jak-Stat signaling (a principal pathway by which cytokines and growth factors control differentiation, proliferation and apoptosis) were found to be aberrant in both EVI1 leukemic cell lines (Figure 3). *Socs1* (supressor of cytokine signaling 1), an inhibitor of STAT transcription factors, was significantly downregulated by 5.7-fold in DA-1 EVI1 leukemic cells, p = 0.01 (Dataset S2), and by 4.4-fold in NFS-60 EVI1 leukemic cells, p = 0.02 (Dataset S3). In NFS-60 leukemic cells, *Stat1* and *Stat5* expression levels were also significantly upregulated (p = 0.02 and p = 0.01 respectively, Dataset S3). Phosphorylation of STAT1 in *Evi1* overexpressed cells was tested in two separate human hematopoietic cell lines with verified *Evi1* overexpression (Kasumi 3 and U937+*Evi1*). Marked increased total STAT1 protein was present in Kasumi 3 cells (an established human leukemic cell line with *Evi1* overexpression) at baseline compared to the control. There was also an increase in phosphorylated STAT1 in Kasumi 3 cells (Figure 4). U937 wildtype and U937+*Evi1* overexpressed cells did not show a marked difference in total STAT1 or phosphorylated STAT1 protein levels (data not shown).

*Osm* (oncostatin M), a cytokine in the interleukin 6 group originally identified to inhibit cell growth in lymphoma cells, was significantly decreased in both DA-1 and NFS-60 leukemic cells (13-fold decrease, p<0.0004 and 1.8-fold decrease, p<0.04, respectively, Datasets S2 and S3). We also identified significant upregulation of *Ube1l* in both cell lines (DA-1 cells 2.5-fold upregulation, p = 0.02, and NFS-60 cells 2-fold upregulation, p = 0.03, Datasets S2 and S3). UBE1L is an activating E1 ubiquitin-like enzyme required for the function of interferon stimulating gene 15 protein (ISG15) [50], a critical modifier of Jak-Stat pathway proteins [51].

**Figure 5:**
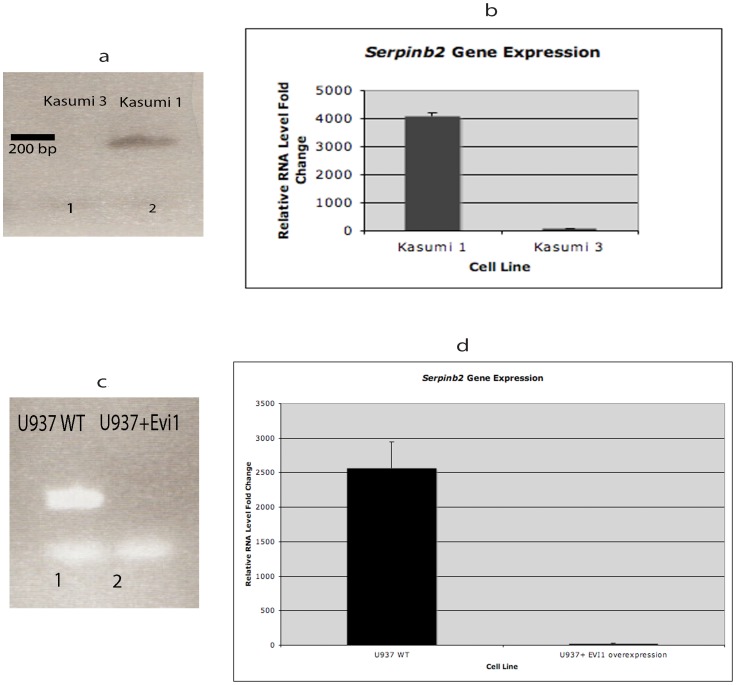
*Serpinb2* downregulation in two human hematopoietic cell lines with *Evi1* overexpression. **a)** Conventional PCR using cDNA from Kasumi 3 with *Evi1* overexpression (Lane 1) and Kasumi1 cells without *Evi1* overexpression (Lane 2). *Serpinb2* is markedly downregulated (no cDNA band detected in PCR) in *Evi1* overexpressed Kasumi 3 human myeloid leukemic cells, consistent with findings in the murine cell lines. **b)** Quantitative RT-PCR shows significant downregulation of *Serpinb2* in *Evi1* overexpressed human leukemic cells. The y-axis value denotes the relative levels of RNA expression based on normalized Ct values. Kasumi 3 cells had >10 point increase in Ct value (less DNA) compared to the Kasumi 1 cells (p<0.0001). **c)** Conventional PCR using cDNA from U937 human leukemic cells. Lane 1 shows U937 wildtype cells (without *Evi1* overexpression) and Lane 2 U937+*Evi1* cells (*Evi1* overexpression). *Serpinb2* is significantly downregulated U937+*Evi1* cells (no cDNA band detected in PCR). d) Quantitative RT-PCR shows significant downregulation of *Serpinb2* in *Evi1* overexpressed human leukemic cells. The y-axis value denotes the relative levels of RNA expression based on normalized Ct values. U937 WT cells had >8 point increase in Ct value (less DNA) compared to the U937+*Evi1* cells (p<0.01).

Several genes associated with cell cycle regulation, specifically those in the serine protease inhibitor (Serpin) family, were significantly downregulated in both EVI1 leukemic cell lines. These included *Serpinb2* and *Serpinf1*. There was a striking 11.4-fold decrease in *Serpinb2* expression in DA-1 EVI1 leukemic cells (Dataset S2), and an 11.5-fold decrease in NFS-60 leukemic cells (Dataset S3). Using conventional and q PCR, we were also able to demonstrate marked *Serpinb2* downregulation in the two human hematopoietic cell lines with *Evi1* overexpression, Kasumi 3 and U937+*Evi1* (Figure 5). *Serpinf1* (also known as pigment epithelium-derived factor, PEDF) was also significantly reduced (DA-1 cells 3.4-fold downregulation, p = 0.02, and NFS-60 cells 2.1-fold downregulation, p = 0.01, Datasets S2 and S3).

Finally we identified several P2X purinoceptors to be significantly downregulated in EVI1 leukemic cells. In DA-1 leukemic cells there was a 6.8-fold decrease in *P2rx2* expression (p<0.05), 21-fold decrease in *P2rx3* (p<0.001), 2.5-fold decrease in *P2rx4*, and 13.6-fold decrease in *P2rx7* (p<0.0003). In NFS-60 cells, there was a 2.0-fold decrease in *P2rx3* expression (Datasets S3 and S4). P2X purinoceptors are ligand-gated ion channel responsible for ATP mediated apoptosis in neutrophils and macrophages [52].

### ChIP-Seq for EVI1 DNA Binding Sites

**Figure 6:**
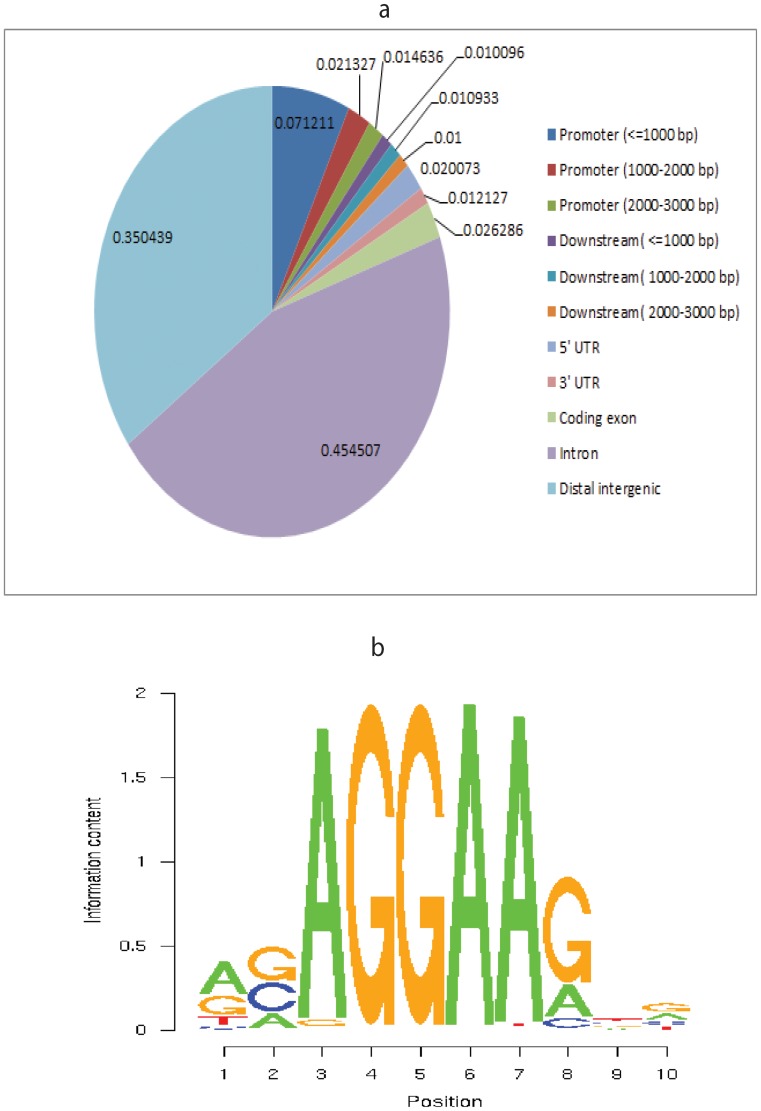
Significant EVI1 DNA binding peaks. Analysis using the UCSC Genome Browser showed the ChIP-Seq EVI1 binding sites demonstrated 10.9% alignment with the whole mouse genome (approximately 5 million reads). 16,745 significant peaks, defined as a difference in number of reads between the control rabbit IgG and EVI1 antiserum antibody yielding a p-value <0.001, were identified based on a Poisson distribution. **a)** Of the 16,745 generated significant peaks (p<0.001), 45.5% were within introns, 35.0% within distal intergenic region, 7.1% were within the proximal (within 1kb) of the TSS. **b)** A 500bp DNA sequence extracted around each significant peak was matched to de novo consensus sequence discovery programs. The AGGAAG ETS-like motif was identified and refined in 88% of the significant EVI1 ChIP-Seq binding sites.

To globally identify direct gene targets of EVI1, we performed ChIP-Seq experiment. DNA bound to EVI1 from the DA-1 murine leukemic cell line was precipitated using both anti C- and N-terminal EVI1 mouse antisera [23]. The generated sequencing reads were mapped to the mouse genome (mm9) by using the bowtie program [53]. This resulted in around 5 million uniquely mapped reads. To identify EVI1 binding peaks, we applied Model-based Analysis of ChIP-Seq (MACS) program [54], which was designed to analyze data generated by short read sequencers such as from the SOLiD platform to first estimate peak size and location, using SAM files as an input. We identified 16,745 significant peaks by using the cutoff of 1.00e-05 for the p-value. We then mapped those peaks on genome-wide scale relative to RefSeq mouse genes (Figure 6a). 7.1% of peaks were within 1kb of the transcription start site (TSS). A de novo motif discovery algorithm, MEME [54], was performed on the top 1000 ranked EVI1 ChIP-Seq peaks. MEME identified an AGGAAG ETS-like motif (E-value = 2.1e-193). We then refined this motif by running TPD [55] all those 16,745 peak regions. Finally, 14,672 out of 16,745 (88%) peaks contained at least one of this ETS-like motif (Figure 6b). Of the 14,672 ChIP-Seq peaks with the AGGAAG ETS-like motif, 4,585 peaks were within promoter regions of an annotated gene (Dataset S4). Our results were consistent with the previously reported EVI1 ChIP-Seq study in ovarian cancer cells which reported 5097 EVI1 significant binding peaks with an ETS-like motif, and over 2000 direct gene targets bound by EVI1 through the ETS-like motif [28].

**Figure 7:**
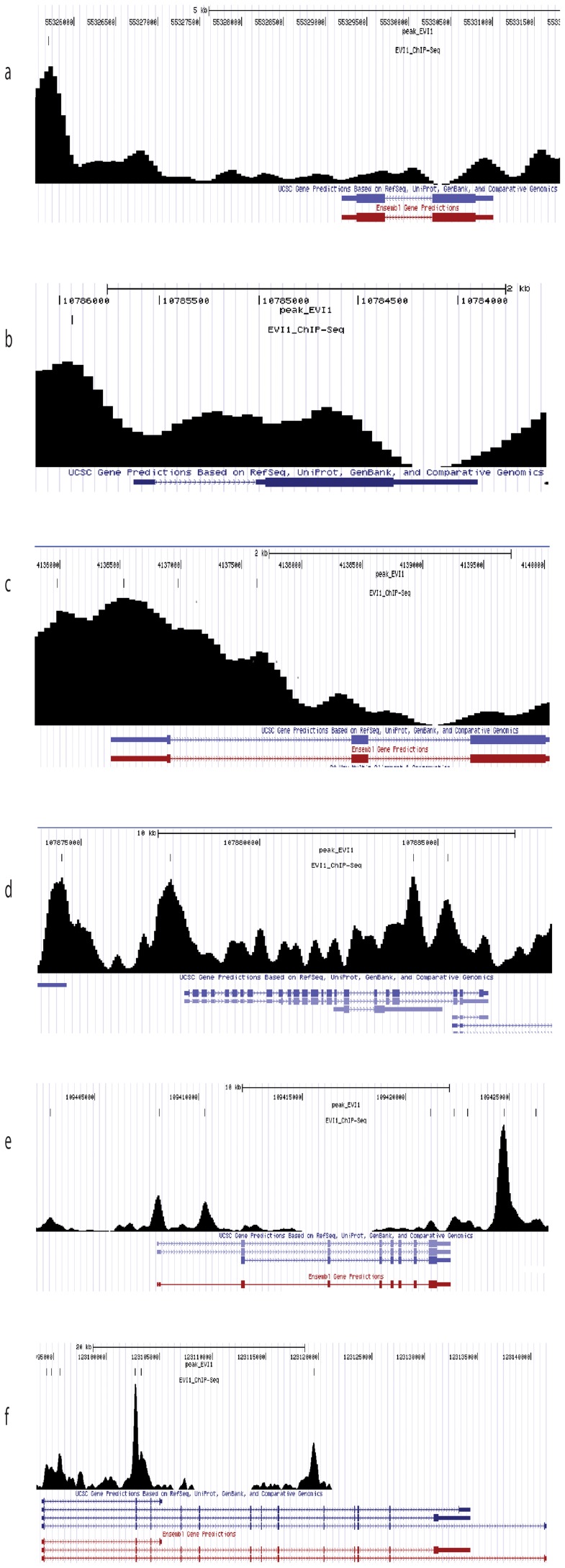
Illustrations of significant ChIP-Seq binding peaks. The UCSC Genome Browser was used to generate significant ChIP-Seq binding peak patterns for *Cebpε*, *Socs1*, *Osm, Ube1l*, *Serpinb2* and *P2rx7* in EVI1 overexpressed leukemic cells.

To provide biological meaning to the significant EVI1 peaks, the Stanford GREAT Analysis Tool was used to assign peaks to nearby annotated genes [56]. EVI1 peaks were significantly associated with 8565 annotated genes (Dataset S5). Of the 35 significantly upregulated and 42 downregulated genes shared by both EVI1 leukemic cell lines, 86% exhibited significant EVI1 DNA binding and deregulation of transcription (Dataset S6). *Cebpe, Socs1* and *Ube1l* were all noted to have significant EVI1 binding. Seven significant EVI1 binding sites were found for *Cebpe* (Figure 7a), 5 with the AGGAAG ETS-like motif and 2 of which were in the promoter region (-2185, −2585 relative to the TSS). Significant EVI1 binding sites were identified for several of the C/EBP family of genes (*Cebpa Cebpb*, *Cebpd*, *Cebpe* and *Cebpg*). However >2-fold changes in gene expression were only present for *Cebpe* in both cell lines (2.2-fold decrease in DA-1 and 2.0–fold decrease in NFS-60, p = 0.02).

Eight significant EVI1 DNA binding sites were identified for *Socs1* (Figure 7b), 7 with the ETS-like motif and 3 of which were within the promoter region (-307, −657 and −1007 relative to the TSS). Significant EVI1 binding sites were also identified for *Socs3* (2 significant binding sites at −5610, −10360 relative to TSS), *Socs4*, *Socs5*, and *Socs7*, but with the exception of *Socs3* in NFS-60 cells, the expression levels for these genes were not significantly different in EVI1 leukemic cells. For *Osm,* 7 significant EVI1 binding sites were found, 6 which were within the promoter region (Figure 7c). Four of the promoter region *Osm* binding sites had the ETS-like binding motif. Two significant EVI1 DNA binding sites were identified for *Ube1l* (Figure 7d), both of which were within the promoter region (-397 and −3447 relative to the transcription start site, TSS) and had the ETS-like motif.

Six significant EVI1 DNA binding sites were found for *Serpinb2* (Figure 7e), all of which had the ETS-like motif, 2 of which were within the promoter region (-1745, −3945 relative to the TSS). Two significant EVI1 DNA binding sites were identified for *Serpinf1* (-6601 and +7384 relative to TSS), both of which had the ETS-like motif.

Regarding genes regulating cellular death, ChIP-Seq revealed 7 EVI1 binding sites for the *P2rx7* gene (Figure 7f), all of which had the ETS-like motif, 3 within the promoter region (+451, +901, +1701 relative to the TSS).

**Figure 8:**
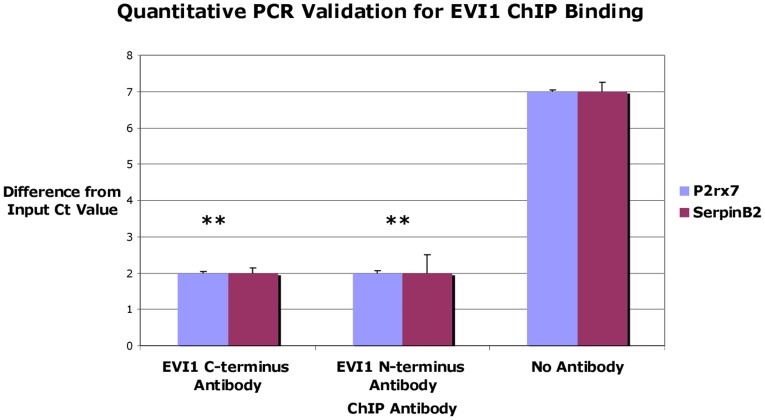
ChIP-qPCR for EVI1 target genes *Serpinb2* and *P2rx7*. ChIP assay was performed using anti-EVI1 C-terminus antisera, anti-EVI1 N-terminus antisera, and a no antibody negative control. The y-axis value denotes the difference in Ct value between the input DNA and sample DNA. The no antibody control had >5 point increase in Ct value (less DNA) compared to the anti-EVI1 DNA samples for both EVI1 target gene primers (p<0.01).

We validated ChIP-Seq peaks for selected genes near or in promoter regions by standard and quantitative PCR analysis using EVI1 antisera and no antibody immunoprecipitated chromatin (Figure 8).

### Shared DNA Binding Sites with Other Transcription Factors

**Figure 9:**
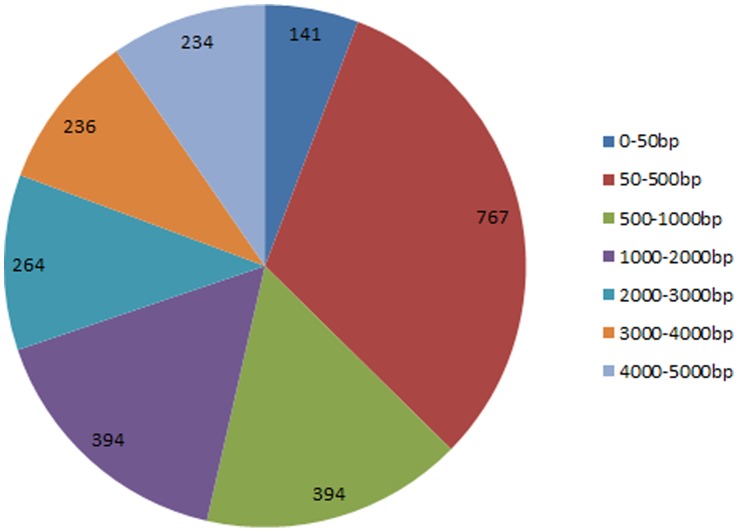
Distribution of significant EVI1 binding sites. The distribution of the 16,745 significant EVI1 ChIP-Seq peaks was plotted against all known transcription start sites (TSS) of annotated genes within the mouse genome using the Stanford Bejerano Lab Great Genomic Regions Enrichment Analyses Tool. EVI1 significantly bound within 5kb of 2,430 annotated genes.

An unusually large number of EVI1 binding sites were identified within 1.5kb of annotated genes, indicating binding within promoter regions and raising the possibility of interactions with other transcription factors (Figure 9). To determine if other transcription factors might bind within the ±1.5 kb regions centered about the annotated EVI1 DNA binding sites, we performed an analysis using the MATCH program and TRANSFAC database [57]. In DA-1 leukemic cells, 79 transcription factors were found to share binding within the promoter regions of EVI1 target genes (p<0.05). In NFS-60 leukemic cells, 67 had shared binding (p<0.05). Sixty two of the same transcription factors were identified to be present in both EVI1 leukemic cell lines. Of these ELK1, an ETS-like transcription factor was found to significantly share binding with EVI1 promoter regions (p = 0.003).

Activator Protein 1 (AP1) was also identified to share EVI1 promoter binding (p<0.03). In a previous ChIP-Seq study in human ovarian cancer cells, AP1 was shown to significantly share EVI1 promoter sites [28]. Other significantly shared transcription factors included NFKβ (p<0.002), PAX4 (p<0.02), PAX5 (p<0.02), and P53 (p<0.03).

### Integrated Functional Pathway Analysis

To determine the important biological pathways involved with genome-wide EVI1 transcription factor binding in *Evi1* overexpressed leukemic cells, DAVID analysis was performed for the 8565 annotated genes significantly associated with EVI1 peaks. The most significant KEGG pathway based on global EVI1 binding were Pathways in cancer (p = 2.5E^−15^), followed by Jak-Stat signaling (p = 2.3E^−11^), Mapk signaling (p = 9.1E^−9^) and Chemokine signaling (p = 1.6E^−7^). Direct EVI1 target genes were also significantly enriched for KEGG pathways specific for Acute Myeloid Leukemia (p = 5.4E^−6^), Apoptosis (p = 3.9E^−4^), Hematopoietic Cell Lineage (p = 3.5E^−5^) and p53 signaling (p = 2.1E^−3^ ).

**Figure 10:**
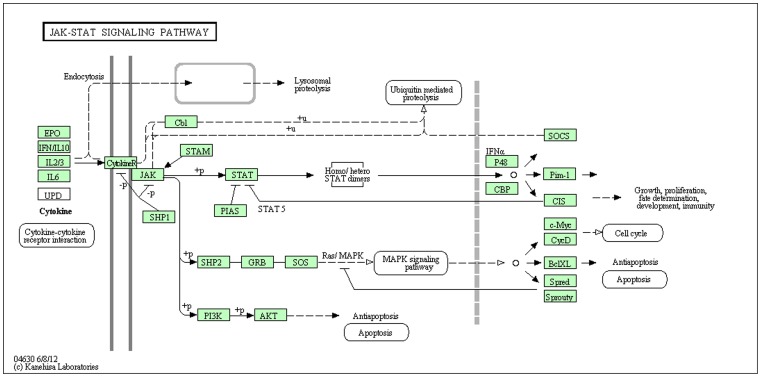
Pathway diagram of the KEGG Jak-Stat pathway using DAVID analysis. EVI1 binds to the majority (78%) of major genes involved in the regulation of the Jak-Stat signaling pathway. These include *Il10, Il10ra, Il6, Il6ra, Cbl, Jak1, Pias2, Stat1, Stat4, Stat5, Stat6, Grb, Sos2, Akt1, Akt2, Socs1,Pim1, Ccnd1, Ccnd2, Ccnd3, Myc,* and *Spred1*. EVI1 binds to an AGGAAG ETS-like motif which is present in the promoter region of all of these Jak-Stat pathway genes. Illustration taken from DAVID website http://david.abcc.ncifcrf.gov/.

DAVID analysis was also performed for the 4,585 annotated genes associated with an EVI1 ChIP-Seq peak with an ETS-like binding motif within its promoter region. Jak-Stat signaling was the most significantly enriched KEGG pathway associated with the annotated genes harboring an AGGAAG ETS-like motif (p<.6E^−7^, Figure 10). EVI1 bound to the promoter regions of 78% of the major genes involved in the Jak-Stat pathway.

Gene set enrichment analysis (GSEA) using curated gene sets from published genomic studies (GSEA Modules C2-C5) was performed to identify distinct molecular signatures for the global EVI1 gene targets. Only genes with significant EVI1 binding sites and deregulation of mRNA transcription were used as input data for the analysis. GSEA revealed these genes were significantly associated with signatures only involving cancer or cancer-oriented genes (C4 Module) (p<0.001, FDR <0.1).

## Discussion

The ecotropic virus integration site 1 (EVI1) is an oncogenic transcription factor associated with a wide range of human malignancies including AML. EVI1 is an independent biomarker that confers poor prognosis in AML. We report here the first genome-wide study of EVI1 DNA binding sites in leukemic cells. We confirmed EVI binding to and deregulation of a select number of previously-reported EVI1 downstream gene targets (Table 3), but not others (Table 4). We also identified novel EVI target genes involved in terminal myeloid differentiation, cell cycle regulation and apoptosis previously unreported in EVI1-induced leukomogenesis. Furthermore, we found the majority of significant EVI1 binding sites contained an ETS-like motif.

**Table 3:** Previously identified EVI1 target genes.

Study	EVI Target Gene (s)	Assay	Present Study	AGGAAG ETS-like Motif Within ChIP Peak	Significant Deregulation in EVI1 Leukemic Cells
Kim et al	*Itpr3*	cDNA hybrid selection	1 peak	(1)	–
Yuasa et al	*Gata2*	ChIP-PCR	15 peaks	(4)	–
Yatsula et al	*2610305D13Rik*	ChIP-PCR	*Dcn* 3 peaks	*Dcn* (0)	*Map3k14* (upregulated in NFS-60)
	*4930503B16Rik*		*Gadd45g* 19 peaks	*Gadd45g* (5)	
	*4930527E24Rik*		*Gata2* 15 peaks	*Gata2* (4)	
	*Dcn*		*Klf5* 2 peaks	*Klf5* (1)	
	*Drd1ip*		*Map3k14* 2 peaks	*Map3k14* (1)	
	*Gadd45g*		*Skil* 3 peaks	*Skil* (3)	
	*Gata2*		*Tnnt2* 1 peak	*Tnnt2* (1)	
	*Gata3*		*Zfpm2* 5 peaks	*Zfpm2* (1)	
	*Klf5*				
	*Map3k14*				
	*Napb*				
	*Plagl1*				
	*Skil*				
	*Tnnt2*				
	*Xmr*				
	*Zfpm2*				
Qui et al	*Calr*	ChIP-PCR	1 peak	(1)	*Calr* (upregulated in DA-1)
Shimabe et al	*Pbx1*	ChIP-PCR	16 peaks	(15)	*Pbx1* (upregulated in DA-1)
Yoshimi et al	*Pten*	ChIP-PCR	9 peaks	(5)	–
DeWeer et al	*Mir449*	ChIP-PCR	None		–
Pradhan et al	*Sirt1*	ChIP-PCR	1 peak	(0)	–
Pradhan et al	*Bcl-xL*	ChIP-ChIP, EMSA	None		–
Bard-Chapeau et al	*Fos*	ChIP-Seq	*Fos* 7 peaks	*Fos* (5)	*Fos* (downregulated in DA-1 and NFS-60)
	*Jun*		*Jun* 6 peaks	*Jun* (6)	*Jun* (downregulated in DA-1 and NFS-60)
	*Gata2*				
	*Numerous others*				

Our study confirmed several EVI1 binding sites near or within previously reported target genes. The number of EVI1 binding sites identified for the specific gene is listed under Present Study. The number of AGGAAG ETS-like motif contained within the ChIP-Seq peaks for the specific EVI1 gene target is in parenthesis. Genes with aberrant expression levels identified by RNA-Seq with bonafide DNA binding by ChIP-Seq in this present study are listed.

**Table 4:** Our study did not identify some previously reported putative EVI1 binding gene targets.

Study	Reported EVI Target Gene (s)	Assay	Present Study ChIP-Peaks
Yatsula et al	*2610305D13Rik*	ChIP-PCR	None
	*4930503B16Rik*		
	*4930527E24Rik*		
	*Drd1ip*		
	*Gata3*		
	*Napb*		
	*Plagl1*		
	*Xmr*		

### *EVI1* Binds and Deregulates a Major Terminal Myeloid Differentiation Gene

C/EBP-ε (CCAAT/enhancer binding protein-ε) is a well established regulator of myeloid lineage differentiation and is critical for the terminal differentiation of granulocytes [58], [59], [60]. Seven significant EVI1 binding sites, 2 of which were within the promoter region, were identified for *Cebpe*. This was associated with a 2-fold downregulation of *Cebpe* in both the *Evi1* overexpressed leukemic cell lines. Unlike other C/EBP family proteins, *Cebpe* expression is restricted to hematopoietic cells, and its activation is associated with terminal differentiation of neutrophils and eosinophils [58]. Koeffler et al demonstrated *Cebpe* knockout mice exhibit neutrophils blocked at the myelocytes and metamyelocytes stage. Clonogenic assays revealed a significant decrease in the number of myeloid colonies, and a significant increase in Lin-Sca1+c-Kit+ colonies [59]. The Yale group showed neutrophils with *Cebpe* knockout have bilobed nuclei, lack secondary granules and mRNA for secondary granule proteins, and exhibit aberrant chemotaxis [61].

As a master regulator of terminal myeloid differentiation, C/EBP-ε binds and activates several downstream gene targets to produce mature granulocytes. To generate a mature neutrophil, a series of committed steps occur from the pluripotent hematopoietic stem cell, which differentiates into the myeloblast, promyelocyte, myelocyte, and finally the band stage. The presence of secondary granules marks the transition from the promyelocyte to the fully committed myelocyte stage [62]. Secondary granule protein genes such as lactoferrin, transcobalamin I, neutrophil collagenase, and neutrophil gelatinase are direct targets of C/EBP-ε [63], [64]. We identified several downregulated C/EBP-ε downstream gene targets in EVI1 leukemic cells. In both *Evi1* overexpressed leukemic cell lines, expression of neutrophil collagenase (*Mmp8*) and neutrophil gelatinase-associated lipocalin (*Lcn2*) were significantly reduced. In the DA-1 leukemic cells, 2 major genes involved in eosinophil maturation (*Epx* and *Prg2*), were also significantly downregulated. We identified at least 6 different downstream C/EBP-ε direct target genes to be downregulated in EVI1-induced leukemic cells. These results suggest it is unlikely that EVI1 directly regulates critical genes involved in myeloid differentiation individually, but binds to and downregulates a master regulator. To our knowledge this is the first report of *Cebpe* deregulation in EVI1-induced leukemia.

### Deregulation of Jak-Stat Signaling in *EVI1* Leukemia

Global biological function analysis using all significant EVI1 binding gene targets revealed the Pathways in cancer and Jak-Stat signaling pathways were most aberrant. Given a surprising 88% of the EVI1 binding sites contained an ETS-like AGGAAG binding motif, we repeated the analysis using only EVI1 gene targets with the motif. This revealed the Jak-Stat signaling was the most significantly enriched KEGG pathway. We found EVI1 significantly binds to the promoter region of a remarkable 50 gene targets involved in the Jak-Stat signaling pathway (Figure 10). Of these 50 genes, expression levels of 10 were significantly aberrant.

Jak-Stat signaling is one of the principal mechanism by which extracellular signals, specifically cytokines and growth factors, are translated into intracellular responses [65]. Various ligands such as erythropoietin, growth hormones, interferons and interleukins bind their cognate receptors which are associated with JAK tyrosine kinases (JAK1, JAK2, JAK 3 and TYK2) [66], [67]. Upon ligand binding, JAKs are transphosphorylated and subsequently phosphorylate latent STAT transcription factors in the cytoplasm. Phosphorylated STATs enter the nucleus and activate or repress gene targets critical for cellular differentiation, proliferation and death [68]. STAT transcription factors are regulated through various inhibitory factors, including the suppressor of cytokine signaling (SOCS) proteins [69].

Excessive Jak-Stat signaling activation results in numerous inflammatory diseases and hematopoietic disorders such as essential thrombocythemia, polycythemia vera, myelofibrosis and leukemias [66], [67]. JAK2 mutations which induce auto-activation of STAT proteins have been well documented in AML [67]. Constitutive activation of STAT 1, 3 and 5 in proliferating human AML blasts have also been reported [70]. We identified *Socs1*, which encodes for an inhibitor of STAT transcription factors, was significantly downregulated by 5.7-fold in DA-1 EVI1 leukemic cells, and by 4.4-fold in NFS-60 EVI1 leukemic cells. We identified 8 significant EVI1 DNA binding sites for *Socs1*, 3 of which were within the promoter region. Two significant EVI1 binding sites were also identified for *Socs3,* but not for *Socs2*. Interestingly, we also found EVI1 significantly binds to and overactivates *Stat1* and *Stat5* genes in one of the *Evi1* overexpressed murine cell lines (NFS-60). We therefore examined if phosphorylated STAT1 protein was increased in two separate human hematopoietic cell lines with verified *Evi1* overexpression (Kasumi 3 and U937+*Evi1*). We found an increased level of endogenous STAT1 protein phosphorylation in Kasumi 3 *Evi1* overexpressed leukemic cells. However, we also noted a marked elevation of total STAT1 protein in these cells, which was consistent with our mRNA findings. Given the baseline level of total STAT1 was much higher in *Evi1* overexpressed leukemic cells, it is unclear at this point if EVI1 directly overactivates Jak-Stat signaling via STAT activation. Although there is a clear interaction between EVI1 and the Jak-Stat pathway, further studies are necessary to elucidate potential mechanisms.

*Osm* (oncostatin M), which encodes for a cytokine in the interleukin 6 family, was also significantly downregulated in our EVI1 leukemic cells. The role of OSM in malignancy remains unclear. Yoshimura et al demonstrated *Osm* is a downstream target of the Jak-Stat pathway [71], transcriptionally induced by cytokines that specifically activate STAT5. OSM has been reported to act as a growth factor in myeloid neoplasms and has also been shown to inhibit proliferation of numerous malignant cell lines, including murine M1 myeloid leukemic cells [72]. OSM also induces differentiation of M1 monocytic leukemia cells and suppresses embryonic stem cell function [73]. We identified 7 significant EVI1 binding sites for *Osm*, 6 which were within the promoter region. EVI1 binding was associated with a significant decrease in transcription in both DA-1 and NFS-60 leukemic cells (13-fold and 1.8-fold decrease, respectively). This suggests downregulation of *Osm* may have an important role in failure of myeloid differentiation in EVI1-induced leukemogenesis.

We also identified a significant increase in *Ube1l* expression in both EVI1 leukemic cell lines (DA-1 cells 2.5-fold and NFS-60 cells 2-fold upregulation). UBE1L is an E1 ubiquitin-like enzyme that is activated at the transcriptional level by type I interferons. UBE1L is required for the conjugation and function of interferon stimulating gene 15 protein (ISG15) [50], a critical modifier of Jak-Stat pathway proteins [51]. *Isg15* is one of the strongest genes induced by type I interferons in response to cellular stress and infection. Upregulation of ISG15 activity has been associated with several cancers [74], [75]. UBE1L E1 enzyme charges ISG15 by forming a thiolester intermediate suitable for transfer to the UBCH8 E2 enzyme [76]. Cong et al demonstrated multipotent hematopoietic progenitor cells from *Ube1L* deficient mice exhibit a G2/M phase block and delay in cellular proliferation, without an effect on survival or differentiation functions [77]. We identified 2 significant EVI1 DNA binding sites for *Ube1l*, both of which were within the promoter region, and associated with a significant increase in *Ube1l* expression in both EVI1 leukemic cell lines. These results suggest EVI1 leukemic cells may harbor sensitivity to cellular stress or inflammatory responses, resulting in uncontrolled cellular proliferation mediated by aberrant UBE1L-ISG15 activation.

### Serpinb2 Downregulation in *EVI1* Leukemia

*Serpinb2*, which encodes for a serine protease inhibitor, was significantly bound by EVI1 and downregulated by >10-fold in both *Evi1* overexpressed leukemic cell lines. *Serpinb2* encodes for plasminogen activator inhibitor (PAI-2), a coagulation factor that inhibits tissue plasminogen activator and urokinase. PAI-2 exists in a secreted, extracellular glycosylated form and an unsecreted intracellular form. PAI-2 is present in monocytes and exists predominantly in the cell cytosol as a 47 kDa nonglycosylated intracellular form [78]. However the intracellular role of PAI-2 is still being established [79].

Some studies report PAI-2 plays a critical role in cell cycle regulation [80], [81]. Nuclear PAI-2 has been shown to bind to the retinoblastoma protein (Rb), a tumor suppressor that prevents excessive cellular division [80]. Inactivation of Rb is associated with malignancy [82]. PAI-2 protects Rb from proteolysis and inhibits its turnover, leading to accelerated Rb-mediated cellular senescence [80]. Monocytes constitutively express PAI-2, but under stress increase *Serpinb2* expression to surprisingly high levels (up to 10,000- fold) [83]. Interestingly, THP-1 monocyte cells do not produce a functionally active PAI-2 protein due to a translocation anomaly [84]. Yu et al demonstrated transfection of wildtype active PAI-2 into THP-1 cells rescues accelerated cellular proliferation [81]. We found significantly decreased *Serpinb2* expression in EVI1 leukemic cells, suggesting it may play an important role in enhancing cellular proliferation by preventing protection of Rb proteolysis.

Alternatively, the decrease in *Serpinb2* expression found in EVI1 leukemic cells may be a marker of reduced differentiation in immature myeloid cells. PAI-2 gene activation has been associated with monocyte differentiation in U-937 monocyte-like cells [85]. Suppressed Serpinb2 expression may be a reflection of EVI1-induced inhibition of myeloid differentiation.

The PAI-2 promoter is tightly regulated under the control of an upstream silencer element (located between −1977 and −1675, termed PAUSE-1) and a repressor element (between −219 and −1100) [86]. We identified a very prominent EVI1 binding site which lies directly within the *Serpinb2* silencer element (position −1745), suggesting EVI1 can potentially disrupt or alter normal binding and function of PAUSE-1 transcription factors. A 67kDa PAUSE-1 BP complex has been shown to bind the silencer element. However, cooperative DNA-binding partners have yet to be identified and may be an area for future study. Additionally, AP1-like elements, AP1a (position −103 to −97) and AP1b (position −114 to −108) have been identified to bind to regulatory elements of *Serpinb2* and induce transcriptional regulation [86]. We have shown EVI1 binds *Serpinb2* to reduce its expression. Bard et al previously demonstrated AP1 physically interacts with EVI1 and frequently shares promoter binding to putative target genes [28]. Collectively, these results suggest the EVI/AP1 may bind *Serpinb2* as a complex to reduce expression and increase cellular proliferation in leukemic cells.

### Disruption of Apoptosis Mediated by Downregulation of ATP-Dependent Purinoceptors

We identified significant downregulation of several genes that encode for ligand gated P2 purinoreceptors, specifically *P2rx3*, *Prx4*, and *P2rx7* in EVI1 leukemic cells. *P2rx7* was of particular interest, given its well established role in regulating apoptosis in macrophages [87], [88], [89]. P2RX7 is a cell surface ATP receptor involved in rapid cell death via calcium influx, and is primarily expressed in macrophages and neutrophils [87]. The ionotropic ligand gated channel is activated by graded doses of ATP which induces reversible permeabilization of the plasma membrane. After channel opening, calcium influx and rapid depolarization [90], [91] leads to a signaling cascade that have been linked to superoxide-mediated mechanisms [89], [92]. Suh et al demonstrated that P2RX7 activation is coupled to the generation of superoxides in human neutrophils [89]. However, the mechanism by which the superoxide production cascade occurs remains unclear.

Previous studies have also shown P2RX7 activation results in release of interferon-1β, accumulation of transcription factors that mediate apoptosis, specifically NFAT and NFKβ [93], [94], and macrophage cell death [87]. P2RX7 activation has also been associated with increased caspase-1 and caspase-3 activity [88]. Caspase inhibitors have shown to inhibit P2RX7-induced NFKβ activity [92]. Humphreys et al demonstrated P2RX7 stimulation with ATP rapidly elevates caspase-3 protease activity associated with DNA fragmentation, and is also strongly linked to upregulation of the c-Jun N-terminal kinase pathway [95]. Failure of apoptosis due to P2 purinoreceptor dysfunction has been implicated in previous studies [96], [97]. We report here that EVI1 binds to 3 sites within the *P2rx7* gene promoter region with significant reduction of *P2rx7* transcription leukemic cells. Our study provides evidence for a potential new mechanism of apoptotic deregulation in the development of AML via ion channel regulation.

### *EVI1* Significantly Binds to an ETS-like Binding Motif

We identified 14,672 ChIP-Seq peaks (88%) with an AGGAAG ETS-like motif. Over 4,500 peaks with this motif were within promoter regions of an annotated gene. Our results are consistent with the only other reported EVI1 ChIP-Seq study, which was performed in human ovarian cancer cells. Their study demonstrated over 5,000 significant EVI1 peaks contained an ETS-like binding motif [28] [28]. The ETS family includes 28 transcription factors in the mouse and has been reported to be important in tissue development and cancer progression [98].

Shared transcription factor analysis revealed the ETS-like transcription factor ELK1, significantly occupied binding sites with EVI1 promoter regions. ELK1 is one of the most studied ETS-like transcription factors [99] and has been implicated in several malignancies, including bladder, breast, esophageal cancers and glioblastoma [99], [100], [101], [102], [103]. Interestingly, a recent ELK1 ChIP-Seq study demonstrated ELK1 binds to redundant DNA regions in cooperation with another ETS-like transcription factor, GABPA 104. However, regions that are occupied by ELK1 but not GAPBA were defined as unique regions associated with gene expression of critical cellular functions. Putative ELK1 competition with GABPA, and potentially other ETS proteins, presents an interesting area for additional study.

**Figure 11:**
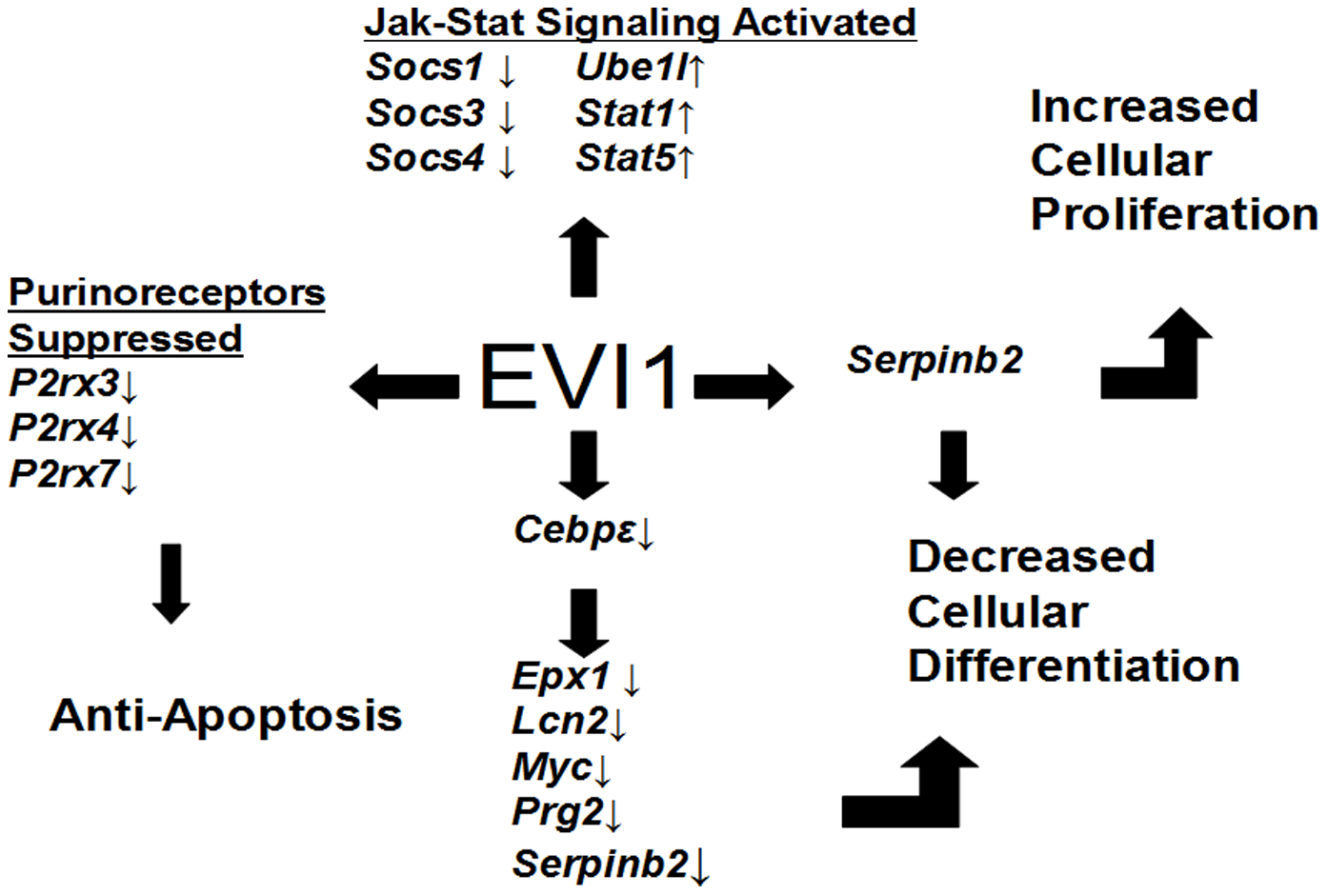
Summary diagram of critical genes involved in EVI1 leukemia.

In summary, these findings represent the first global genome-wide study of EVI1 DNA binding associated with whole transcriptome expression analysis. Our results reveal several important genes with an ETS-like binding motif, is involved in terminal myeloid differentiation, cell cycle regulation and apoptosis [Figure 11]. The Jak-stat pathway and response to inflammatory and stress conditions were notably aberrant. We have previously shown that small molecule inhibitors against EVI1 gene targets can be designed to successfully block its binding [20]. This study provides a list of critical genes that can be targeted for future anti-leukemic therapies. We demonstrate that several gene targets work in concert to drive leukemogenesis. This suggest a cocktail of inhibitors targeting a select number of DNA sites, rather than a drug targeting an isolated gene, may be a more promising approach for developing a cure for EVI1-induced leukemogenesis.

## Materials and Methods

### shRNA Suppression of *Evi11* and RNA-Sequencing

*Evi1* expressing AML cell lines (DA-1 [105]and NFS-60 [106]) were transduced with retrovirus expressing EVI1 targeting shRNAs (sh11 and sh54) and OFF-targeting shRNAs (shScr and shLuc). After 48 hrs cells were selected for puromycin-resistance for 48 hrs and purified on Ficoll-Paque PLUS gradient (GE healthcare Bio-Sciences AB; Uppsula Sweden). Ficoll-buoyant cells were harvested and Western blot analyses of protein extracts from DA-1 and NFS-60 cells transduced with control (shScr) or anti-*Evi1* (sh11) was completed using anti-EVI1 serum. Total RNA was prepared with Qiagen RNeasy plus kit with QIAshredders (Qiagen Sciences; Maryland). Quantitative RT-PCR analysis of *Evi1* transcript levels in DA-1 and NFS-60 cells were performed following retroviral infection with the shRNA constructs. 200 ng of RNA per sample were processed with Illumina TruSeq RNA High-Throughput sample preparation protocol (Illumina,Inc; SanDiego, CA) and analyzed on Genome Analyzer *IIx* (Illumina,Inc; SanDiego, CA).

### *Evi1* Overexpression in Human Hematopoietic Cell Lines

**Figure 12:**
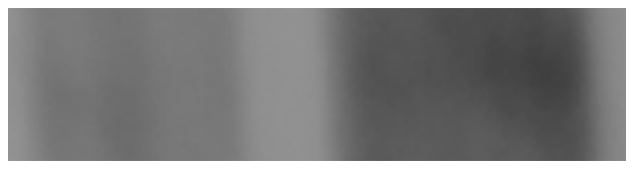
Western blot to confirm EVI1 overexpression. T293 cells were transfected using the Gag Pol and VSV viral packaging system. Forty-eight hours post tranfection the viral supernatant was used to infect U937 wildtype cells. This figure shows the Western blot to confirm overexpression of EVI1 in U937 cells. Lane 1 from the left shows no EVI1 band in U937 wildtype cells while lane 2 represents EVI1 expression in transfected U937 cells with *Evi1* construct.

Kasumi 3 cells, an established human AML cell line with 3q27 translocation and *Evi1* overexpression were purchased from ATCC® and expanded in RPMI-1640 media with 10% FBS. Kasumi 1 cells, a leukemic cell line with 8q21 translocation and without *Evi1* overexpression was used as a control to demonstrate downregulation of significant genes. U937 histiocytic lymphoma cells purchased from ATCC® were retrovirally transduced with an *Evi1* construct to overexpress EVI1. T293 cells were transfected using the Gag Pol and VSV viral packaging system. Forty eight hours post tranfection the viral supernatant was used to infect U937 wildtype cells. A Western blot was performed to confirm overexpression of EVI1 (Figure 12). U937 cells with *Evi1* overexpression were sorted using flow cytometry using the green fluorescent protein selection marker.

### Western Blot in Human Leukemic Cells

Immunoblot analysis using the EVI1 C-terminal antibody was performed. Approximately 3 million cells were lysed in ice cold RIPA buffer with protease and phosphatase inhibitors. A Bradford assay was used to calculate protein concentrations for each sample (Kasumi 1, Kasumi 3, U937 wildtype. U937+ *Evi1*). Samples were diluted in 5X SDS buffer for equal concentration gel loading. Beta actin was used as a gel loading control (Figure 4). Protein transfer to a nitrocellulose membrane was completed as previously described. Primary antibodies included anti-phosph-STAT1 (Cell Signaling®, 1∶1000 dilution), anti-STAT1 (BD Transduction®, 1∶1000 dilution) and anti-beta-actin (Cell Signaling®, 1∶1000 dilution). Conjugated HRP sheep-anti mouse and donkey-anti rabbit secondary antibodies were used (GE Healthcare®). The Biorad® ChemiDoc™ XRS+ System and Image Lab software were used to detect chemiluminescence.

### ChIP-Seq and PCR

ChIP was completed using a lyophilized Staph A Cell (Pansorbin®) Assay Protocol as previously described [107]. Antisera specific for the N- and C-terminus of the murine EVI1 protein (GenBank™ accession number M21829) were generated using purified protein as previously described [23]. Immunoprecipitation was performed using EVI1 antisera, with no antibody and rabbit IgG as negative controls. Confirmation of genomic DNA for selected EVI1 binding target genes was completed with quantitative PCR reactions using the appropriate positive and negative control primers (Table 5). Input DNA libraries were prepared and sequenced using the SOLiD™ System (Applied Biosystems) platform [53], [54]. PCR reaction primers for cDNA expression in human Kasumi1, Kasumi3, U937 wildtype and U937+*Evi1* are listed in Table 6.

**Table 5:** Quantitative PCR primer sets used to confirm novel EVI1 target genes.

Gene	Forward Primer	Reverse Primer
*Cebpe*	GCC GAG CTT ATC TCC CA	GGA AAT CCC TAT CAC CAC
*P2rx7*	GAC TGT CAC CAG CAG CA	GGA GCT GAT AAC AGG CT
*Serpinb2*	GCA CGA TAT GCT GTC AT	TAC TCC AGG AAG GAA GAG

**Table 6:** PCR primer sets used to confirm significantly downregulated cDNA expression levels in *Evi1* overexpressed human cell lines.

Gene	Forward Primer	Reverse Primer
*Cebpe*	AGCTAGGGGACATGTGT	TGGAGGGTAGGCAAA
*Serpinb2*	CCAAGCCATGGTGGATGTG	TGGGCCTCCATGTCCAGTT
*Beta Actin*	TGGATGATGATATCGCC	ATGCCTCTCTTGCTCTG

### Computational and Functional Analysis

The ChIP-Seq read were mapped to a reference genome by bowtie program, allowing two mismatches. Multiple aligned reads were removed from the analysis. The aligned sequence reads were displayed as a track onto the mouse reference genome using the University of California at Santa Cruz (UCSC) genome browser (http://genome.ucsc.edu/index.html) for visual inspection. To determine where the EVI1 bound to the genome, we applied the MACS program to look for areas where there were significantly more enriched reads mapped in the ChIP sample. The Stanford Genomic Regions Enrichment of Annotations Tool (GREAT v1.2.6) was used to assign significant peaks (non-coding genomic regions) to annotated genes *in cis* [56]. A 500bp DNA sequence was extracted around each peak and run against MEME and TPD programs to identify potential EVI1 consensus binding motifs. The MATCH program in the TRANSFAC database was used to perform the search for the enrichment of other transcription factors binding sites (TFBS) in the ±250 bp regions centered on the annotated EVI1 binding sites. We set up a filtering step utilizing the cross-species conservation information. The conservation score for each TFBS was evaluated to be the average phastCons score within the TFBS region. A cut-off of 0.5 for the conservation score was applied here and Fisher’s exact test was used to detected statistical significance.

Integrated functional pathway analysis using DAVID and GSEA were completed for gene lists generated from significantly up and downregulated transcripts with EVI1 DNA binding sites. For GSEA, a hypergeometric distribution was used to determine enrichment score (FDR <0.10) when compared to curated GSEA gene sets C2-C5. C2-C5 collection of gene sets included data from over 340 PubMed articles and online databases from Biocarta, Gene array, BioScience Corp, KEGG, Reactome, Sigma-Aldrich Pathways, Signal Transduction Knowledge Environment and Signaling Gateway [108].

## Supporting Information

Dataset S1 **RNA-Seq data in** ***Evi1*** **overexpressed myeloid leukemic cells (DA-1, NFS-60) and in shRNA** ***Evi1*** **knockdown cells.** To identify genes differentially expressed between *Evi1* overexpressed myeloid leukemic cells (DA-1, NFS-60) and in shRNA *Evi1* knockdown cells, RNA was extracted to generate transcriptome-wide expression profiles. High throughput parallel sequencing revealed 806 significantly deregulated (p<0.05) genes in DA-1 cells (481 upregulated, 325 downregulated in the *Evi1* overexpressed cells compared to the *Evi1* shRNA knockdown) and 782 deregulated genes in the NFS-60 cell line (437 upregulated, 345 downregulated).
(XLS)

Dataset S2 **Genes differentially expressed between DA-1** ***Evi1*** **overexpressed myeloid leukemic cells and in shRNA** ***Evi1*** **knockdown cells,** In DA-1 *Evi1* overexpressed leukemic cells, a high number (N = 6) of significantly downregulated direct gene targets of C/EBP-ε were identified.
(XLS)

Dataset S3 **Genes differentially expressed between NFS-60** ***Evi1*** **overexpressed myeloid leukemic cells and in shRNA** ***Evi1*** **knockdown cells,** In NFS-60 leukemic cells, 3 C/EBP-ε direct gene targets were also significantly downregulated. These results demonstrate EVI1 leukemic cells induce downregulation of *Cebpe* expression, but also repress downstream target genes of the master differentiation regulator.
(XLS)

Dataset S4 **EVI1 binds within the promoter region of an annotated gene.** MEME identified an AGGAAG ETS-like motif (E-value = 2.1e-193). Eighty-eight percent of significant peaks contained at least one of this ETS-like motif. Of these, 4,585 peaks were within promoter regions of an annotated gene.
(XLS)

Dataset S5 **The Stanford GREAT Analysis Tool (Bejerano Lab).** To provide biological meaning to the significant EVI1 peaks, peaks were assigned to nearby annotated genes and associated with 8565 genes.
(XLS)

Dataset S6 **Deregulated genes in both DA-1 and NFS-60 cell lines.** Of the 35 significantly upregulated and 42 downregulated genes shared by both EVI1 leukemic cell lines, 86% exhibited significant EVI1 DNA binding and deregulation of transcription.
(XLS)
